# The extramural metastasis might be categorized in lymph node staging for colorectal cancer

**DOI:** 10.1186/1471-2407-11-414

**Published:** 2011-09-26

**Authors:** Hai-Bo Qiu, Gong Chen, Rajiv P Keshari, Hui-Yan Luo, Wang Fang, Miao-Zhen Qiu, Zhi-Wei Zhou, Rui-Hua Xu

**Affiliations:** 1 State Key Laboratory of Oncology in South China, Guangzhou, Guangdong, 510060, P. R. China; 2 Department of Medical Oncology, Sun Yat-sen University Cancer Center, Guangzhou, Guangdong, 510060, P. R. China; 3 Department of Gastric & Pancreatic Surgery, Sun Yat-sen University Cancer Center, Guangzhou, Guangdong, 510060, P. R. China; 4 Department of Colorectal Surgery, Sun Yat-sen University Cancer Center, Guangzhou, Guangdong, 510060, P. R. China; 5 Department of Pathology, Sun Yat-sen University Cancer Center, Guangzhou, Guangdong, 510060, P. R. China

**Keywords:** extramural metastasis, staging, colorectal cancer

## Abstract

**Background:**

The objective of this study is to assess the clinical significance and prognostic impact of extramural metastasis in colorectal carcinoma and establish an optimal categorization in the staging system.

**Methods:**

To determine the frequency and prognostic significance of extramural metastasis, from 2000 to 2005, a total of 1,215 patients with colorectal cancer who underwent surgical resection were recruited into this study. Individual demographic and clinicopathologic data were collected including tumor stage, nodal stage, tumor histology, degree of tumor differentiation, and presence of lymphovascular invasion. After surgery, all patients received standard treatments and follow-up, which were closed in April 2010.

**Results:**

EM was detected in 167 (13.7%) patients and in 230 (1.8%) of the 12,534 nodules retrieved as 'lymph nodes'. The incidence of extramural metastasis was significantly higher in patients with large tumors, deeper invasive depth and more lymph node metastasis (P < 0.001). After curative operation, overall survival was significantly worse for patients with extramural metastasis than those without (P < 0.001). Multivariate analysis identified extramural metastasis as an independent prognostic factor (RR = 2.1, 95%CI:1.5-3.0). By using the Akaike information criterion (AIC), N staging was capable of predicting survival outcome with the highest accuracy when both nodal involvement and extramural metastasis were treated together as N factors(AIC = 1025.3).

**Conclusion:**

Extramural metastasis might be diagnosed as replaced lymph nodes in the process of classification, thus forming a new categorization.

## Background

Histologic examination of dissected nodal structures may disclose the presence of nodules of tumors that are not contained within recognizable lymph nodes. This kind of cancer deposit called Extramural Metastasis (EM), is found during routine examination of about 10-20 percent of resected gastric carcinoma specimens [1] and 15-50 percent of colorectal carcinoma specimens [2,3]. The presence of EM has also been identified as a prognostic factor [2], but whether the EM should be treated as equal to other traditional prognostic factors is still unknown.

The TNM classification is used worldwide for cancer staging. It's important that the patients' treatment and prognosis be followed and built upon this classification. EM may be either small lymph nodes or lymphoid aggregates that have been totally replaced by a tumor or discontinuous foci of a tumor within a pericolonic, extranodal, perivascular or intravascular location [4]. It is unclear whether EM should be categorized into pT staging, pN staging or excluded from consideration in determining tumor stage. Based on the 5^th ^edition TNM Classification of Malignant Tumors [5], a tumor nodule greater than 3 mm in diameter is classified in the N category as lymph node metastasis. The 6^th ^edition of the TNM classification [6] recognizes the heterogeneity of these lesions and suggests that they be classified as positive lymph nodes if they occur in the connective tissue of a lymph drainage area and have the form and smooth contour of a lymph node; if they have an irregular contour, then they should be classified under T as a discontinuous extramural extension. However, some challenge this because they believe size should not affect the diagnosis of metastatic cancer and that tumor foci which show evidence of growth(eg. glandular differentiation, distension of sinus, or stromal reaction) should be diagnosed as a lymph node metastasis regardless of size [7]. Recently, the 7^th ^edition of the TNM classification has been modified such that the extramural tumor deposits found in patients with T1 and T2 lesions be classified as N1c disease [8], but it is still unclear how to stage the EM for patients with T3 or T4. To date, few studies have discussed the significance of the EM through convincing analysis in colorectal cancer, and there are even less data on the optimal categorization of such foci. Nevertheless, an optimal categorization should heighten the value of the TNM classification as a prognostic grading system.

Therefore, the aim of the present study is to assess the incidence and extent of EM in patients with colorectal cancer. Furthermore, its relation to other clinicopathologic factors was studied and its prognostic significance was analyzed. To resolve the question of their origin, we classified them into several different stages based on our provisional definition of the EM and determined whether EM should be categorized in the prognostic staging system, providing useful prognostic information to optimize the TNM classification.

## Methods

### Patients

In this retrospective study, data were collected from the medical records of 1,215 patients who underwent resection for colorectal cancer at the Sun Yat-Sen University Cancer Center in Guangzhou, China, from January 2000 to December 2005. To be eligible for the study, patients were required to have pathologically confirmed colorectal carcinoma and had more than 12 lymph nodes from surgical specimens. All patients underwent standard segmental colectomy and regional lymphadenectomy. Individual demographic and clinicopathologic data were collected including tumor stage, nodal stage, tumor histology, degree of tumor differentiation, and presence of lymphovascular invasion. The protocol was approved by our institutional review board in keeping with Chinese bioethical regulations. All patients gave a written informed consent before participating in the study.

EM was defined as the presence of cancer cells in soft tissue that was discontinuous with the primary lesion or in peri-bowel soft tissue distinct from the lymph node. Lymph nodes and EM were identified and retrieved from formalin-fixed surgical specimens without using any specific technique to increase the nodal retrieval rate. Paraffin-embedded specimens were stained with haematoxylin and eosin, and examined microscopically for metastases. All cases with clolorectal cancer were subsequently step-sectioned, resulting in a total of 119,070 slides, which were H&E stained and systematically screened by 2 pathologists independently. No special immunohistochemical techniques were used to identify micrometastases.

### Treatment

Eligible patients had completely resected primary colorectal adenocarcinoma. The staging was according to AJCC Cancer Staging Manual 6^th ^edition [6]; Based on this staging, all the patients who need adjuvant chemotherapy received the 5-FU-based regimen, and the basic regimen was *FOLFOX6 *or *XELOX*. The patients with rectal cancer received prior chemotherapy or radiotherapy and the patients with post-operative metastasis underwent palliative treatments (including chemotherapy, surgical resection and radio frequency ablation) according to the updated NCCN guideline for Colorectal cancer [9].

### Follow up

After discharge from the hospital, all patients entered a follow-up program according to standard protocol [10]. Within the first 2 years after surgery, a follow-up every 3 months consisted of a clinical examination, routine blood tests, assessment of concentration of tumor markers, and abdominal ultrasonography or CT scan; endoscopy was done every 6 months for the first 2 years after surgery. In addition, for the next 3 years, patients were followed up every 6 months and underwent endoscopy every 12 months. At relapse (defined as local recurrence or metastasis at distant sites), all patients were staged fully to detect disease at other sites. The follow-up was closed in April 2010.

### Statistical Analysis

In this study, all patients had been retrospectively reclassified into different stages according to AJCC Cancer Staging Manual 7^th ^edition. Mann-Whitney U and χ2 tests were used where appropriate to compare the distribution of individual variables between groups. Overall survival (OS) and Disease-free survival(DFS) curves were calculated by Kaplan-Meier method and the differences between 2 groups were compared by log-rank test. The probability for entering the model was 0.05 and that for removal from the model was 0.100. Multivariable analysis was performed using a Cox proportional hazards model with an enter procedure. A two-tailed p value of less than 0.05 was considered statistically significant. The Akaike Information Criterion (AIC) [11] was used to identify the optimal categorization of EM that afforded the N and T stages the highest power of discrimination of survival outcome. The AIC (AIC = -2 × Log Likelihood + 2 × No. of Parameters in the Model) is an estimate of the measure of fit of a model to a given set of data. The model of choice achieves parsimony with maximum likelihood and is the one with the lowest value of AIC, indicating the smallest loss of information for predicting outcome [12]. Statistical analysis was performed with SPSS for Windows V.13.0.

## Results

**Figure 1:**
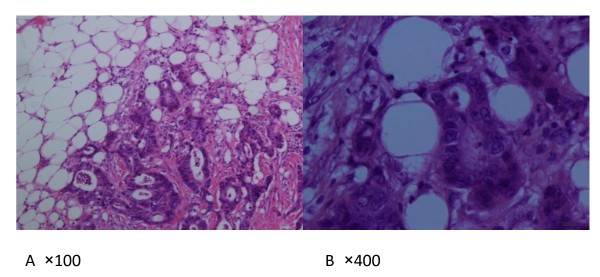
**Haematoxylin and eosin staining shows extramural metastasis in colorectal carcinoma**. Tumor cells are scattered into the peri-bowel soft tissue distinct from the metastatic lymph node. A Original magnification × 100, B × 400.

The study consisted of 695 men and 520 women, the median age was 58 years (range 18-85). Stage distribution included 210(17.3%) patients with stage I, 395 (32.5%) patients with stage II, 336(27.7%) with stage III and 274(22.6%) with stage IV. Overall, EM(Figure 1) was detected in 167 (13.7%) of the 1215 patients and in 230 (1·8%) of the 12,534 nodules retrieved as 'lymph nodes'. In the 167 patients with EM, the mean number of metastases of this type was 1.4 (median 1, range 1-5). Figure 1 shows an example of EM.

The incidence of EM was significantly higher in patients with large tumors (diameter 5 cm or more) and in those with macroscopic infiltrative tumors. Histologically, the incidence of EM had positive correlation with tumor penetration depth and lymph node metastasis, which were determined by leaving the existence of EM out of the calculation. Patients with EM had significantly deeper tumor invasion and larger number of lymph node metastases; in addition, peritoneal dissemination was found more frequently at surgery in patients with EM (Table 1).

**Table 1:** Correlation between EM and clinicopathological features in Colorectal Cancer

Factors	Extramural Metastasis(EM)		P-value
	
	Positive (n = 167,%)	Negative (n = 1,048,%)	
Age(years)			0.093
< 58	94(56.3)	520(49.6)	
≥58	73(43.7)	528(50.4)	
Gender			0.677
Male	98(58.7)	597(57.0)	
Female	69(41.3)	451(43.0)	
Tumor size			0.003
< 5 cm	88(52.7)	676(64.5)	
≥ 5 cm	79(47.3)	372(35.5)	
Location			0.100
Colon	73(43.7)	530(50.6)	
Rectum	94(56.3)	518(49.4)	
Differentiation			0.411
Well	16(9.6)	89(8.5)	
Moderately	111(66.5)	754(71.9)	
Poorly	40(24.0)	205(19.6)	
Invasive Depth			< 0.001
T1	3(1.8)	58(5.5)	
T2	19(11.4)	195(18.6)	
T3	45(26.9)	339(32.3)	
T4	100(59.9)	456(43.5)	
Lymph Node Metastasis			< 0.001
N0	71(42.5)	626(59.7)	
N1	55(32.9)	266(25.4)	
N2	41(24.6)	154(14.7)	
Peritoneal metastasis			0.032
Yes	21(12.6)	80 (7.6)	
No	146(87.4)	968(92.4)	
Liver metastasis			0.232
Yes	27(16.2)	134(12.8)	
No	140(83.8)	914(87.2)	

936 (77.0%) of all patients and 112 (67.0%) of 167 patients with EM underwent curative surgery. After a potentially curative procedure, 66(58.9%) of 112 patients with EM experienced recurrence, compared with 187 (22·6%) of 824 patients without EM. Of those classified as EM, 23 patients (34.8%) had recurrence locally or in the lymph nodes, 21 patients (31.8%) had hepatic involvement and 16 (24,2%) had peritoneum metastases. The primary site of recurrence was unknown in 6 patients.

**Figure 2:**
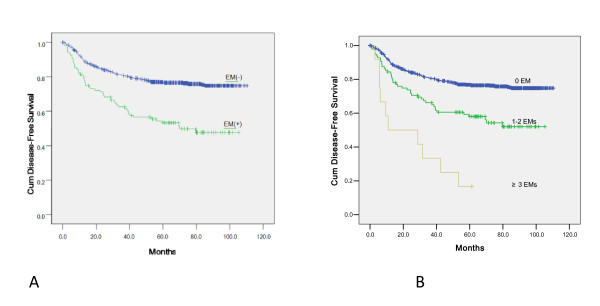
**Survival analysis of EM in patients with colorectal cancer**. A: Prognostic significance of Extramural Metastasis(EM) on Disease-Free Survival(DFS) of the CRC patients underwent curative surgery(p < 0.001), B: DFS curves of CRC patients stratified by EM number (0, 1-2, ≥3) (p < 0.001).

Regarding survival, analysis was performed in 936 patients who underwent potentially curative resection; traditional prognostic variables such as the pT, pN, tumor differentiation and tumor site correlated well with the patients' life expectation (Table 2), with tumor stage according to the TNM system being a strong predictor of survival. A correlation with poorer clinical outcome was also noted for poorly differentiated and rectum location (data not shown). In the multivariate analysis, EM emerged as an independent prognostic factor for survival (Table 2) together with the aforementioned factors. A positive EM at any site was significantly associated with a shorter survival time (Figure 2a). Analysis of patients grouped according to the number of EMs revealed that the number of metastases was significantly associated with an even worse prognosis (P < 0·001) (Figure 2b).

**Table 2:** Univariate and multivariate Cox proportional hazard model

Variable	Univariate			Multivariate		
	
	P Value	RR	95%CI	P Value	RR	95%CI
EM	< 0.001	2.4	1.8-3.3	< 0.001	2.1	1.5-3.0
Tumor site	< 0.001	1.5	1.1-1.9	0.027	1.4	1.0-1.8
Differentiate	0.007	1.5	1.1-2.0	0.044	1.4	1.0-1.8
pT stage	0.002	1.3	1.1-1.5	0.033	1.3	1.1-1.5
pN stage	< 0.001	2.0	1.7-2.4	< 0.001	1.7	1.4-2.0

Different survival outcomes based on the N stage (N0, N1, and N2) and the T stage (T1, T2, T3 and T4) were compared among the following 3 definitions of the N and T categories: A, 7^th ^edition the TNM definition without considering EM; B, 7^th ^edition the TNM definition with EM considered as a T factor (T3 or T4); C, 7^th ^edition the TNM definition with EM considered a N factor (Table 3).

**Table 3:** Definitions of N and T Categories and Their Impact on the Prognostic Value of N and T Staging Systems

Definition	EM Classified into N and T Categories		N Staging		T Staging	
	**N**	**T**	**AIC**	**HR (95% CI)**	**AIC**	**HR (95% CI)**

A	TNM classification (7th ed)	1040.2	2.37(1.92-2.92)	1094.1	1.36(1.15-1.60)	
B	Distinct nodal involvement	EM for (T3 or T4)	1040.2	2.37(1.92-2.92)	1089.7	1.42(1.20-1.68)
C	1.Distinct nodal involvement	pT staging	1025.3	2.65(2.15-3.27)	1090.9	1.37(1.15-1.61)
	2.EX					

**Figure 3:**
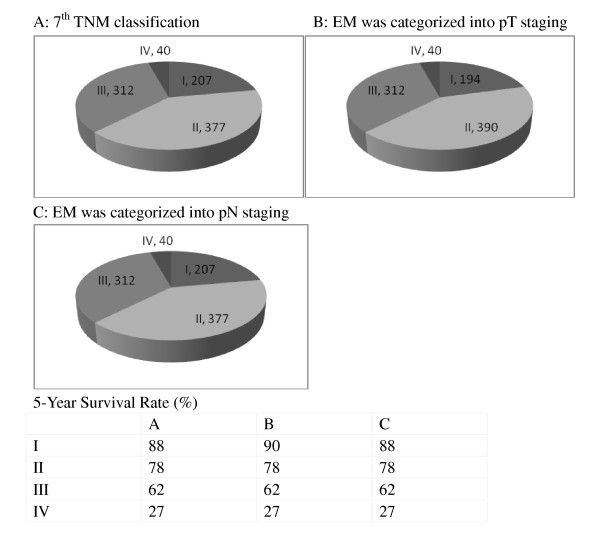
**Distribution and 5-year survival rate of 936 patients based on the EM stratified into four different categories**. A: 7th TNM classification, B: EM was categorized into pT staging, C: EM was categorized into pN stagin.

According to the value of AIC, for the N stage, the definition that both distinct nodal involvement and EM were treated as N factors has the lowest ability(AIC = 1025.3) under the condition of using the 7th edition TNM classification. For the T stage, the AIC in the group that EM were treated as pT3 or pT4, is the smallest(AIC = 1089.7), which means it provided the best prognostic T staging. The AIC in the group that has both distinct nodal involvement and EM treated as N factors also has a good value (AIC = 1090.9). Distribution and 5-year survival rate of 936 patients based on the EM stratified into 3 different categories is summarized in Figure 3; the difference in 5-year survival between these defined groups was not great, and the differences in the survival between different T staging under the aforementioned provisional definitions were much less than those between the values of the N stage.

## Discussion

In this retrospective study, we investigated the clinical parameters and the prognostic value of EM in a group of patients who underwent surgical resection for colorectal carcinoma. The main findings are the following: (1) the presence of EM had a significant correlation with the primary tumor size, invasive depth and lymph node metastasis; (2) the patients with EM had a higher risk of peritoneal metastasis, EM is an independent prognostic factor for all colorectal cancer patients, and the patients with EM had a very poor clinical outcome; and (3) EM may be regarded as lymph nodes in the process of classification using 7^th ^AJCC Cancer Staging Manual.

Metastatic status determines prognosis and indication for adjuvant chemotherapy in patients with colorectal cancer [13]. A recent population-based study in the United States concluded that, in 2001, most patients with colorectal cancer received inadequate evaluation [14]. There is, thus, an important risk of understaging that may exclude patients from postoperative chemotherapy, a treatment with proven benefits in colon cancer with advanced stage [15]. The existence of isolated tumor deposits in the mesorectum was well demonstrated by microscopic examination with serial transverse sections of the mesorectum made as reported by Scott et al [16] and Reynolds et al [17]. In routine practice, such tumor deposits are observed in specimens sent for pathologic examination as "lymph nodes." There is few study focus on these kinds of "lymph nodes" without lymph node structure. The present study comprised patients with colorectal cancer who underwent surgical resection. We found that the presence of EM was about 15%, which was associated with a larger tumor size, deeper invasion depth and more positive nodes; this finding showed a close correlation with cancer aggressiveness measured in terms of serosal invasion. It has clinical significance as a strong prognostic indicator independent of tumor depth or nodal involvement in patients with colorectal cancer who underwent operation, though we cannot neglect the effect of EM on the survival outcome in the patients with colorectal cancer.

In the incidence of peritoneal metastasis found during surgery in patients with EM, our results were similar to Etoh et al. [2]. There are two possible explanations for the association between EM and peritoneal metastasis. First, it is feasible that tumor cells released from a primary lesion spread directly into the extranodal and extramural spaces [1,18]. Another possibility is that EM occurs subsequent to lymph node involvement. Takata et al. [1] found that the occurrence of lymph node metastasis was high in patients with peritoneal metastasis. EM was associated with peritoneal metastasis, which is one of the most important prognostic factors in this tumor type; the patients with EMs had a shorter OS and DFS. The multivariate analysis shows that EM is an independent prognostic factor along with pT, pN staging, tumor site and tumor differentiation. In addition, the numbers of EMs have an important role in the estimation of postoperative survival. The existence of 3 or more EMs might have a poorer prognostic impact than those with 1-2 deposits of EM; in the present study, 3 or more EMs were found in 4.3% of patients who had undergone curative resection of colorectal cancer and related to an unfavorable 5-year survival rate of 18.0%. Our findings confirming that EM is a negative prognostic factor for advanced colorectal adenocarcinoma are helpful to those clinicians in need of a staging system which emphasizes the prognostic heterogeneity of patients within the same tumor stage group. The different prognostic implications of EM could well be incorporated into new staging proposals. It is very important to determine whether EM should be treated as an N factor or as a T factor in a prognostic staging system, so that the clinician can more conveniently provide an accurate classification.

We compared 3 staging systems with different categorizations of EM, including categorizations based on the TNM classifications of the 7th editions in terms of their discriminatory power with regard to survival outcome. In our analysis, we used the AIC which can be used to identify the optimal categorization of an outcome variable and to compare systems with different combinations of variables [19], although in the difference in the 5-year survival rate of patients classified according to the various staging systems shown in Figure 3, we found that the value of AIC was smallest when both distinct nodal involvement and EM were treated as N factors. We still note that there was little difference in the AIC value among the T staging systems analyzed, and it could be said that the classification of EM exerts little influence on the clinical significance of the T stage because of the low incidence of EM in T1 and T2 cases, which indicated that the EM might be treated as an N factor defined by the TNM 7th edition. To our knowledge, our results initially report this new categorization.

In terms of the new categorization, the number of patients shows little difference from other various staging systems (Figure 3), but the clinically most beneficial staging system is one that assigns as many patients as possible to the most favorable or most unfavorable stage when survival outcome of patients classified in the same stage is the same [20]. Ueno et al. in 2007 [21] proposed that N staging was capable of predicting survival outcome with the highest accuracy when both nodal involvement and non-vascular invasion-type (non-VAS) were treated together as an N factor and VAS was treated as a T factor, but the use of the subjective description form and smooth contour of a lymph node may lead to inappropriate upstaging [7]. The current dilemma can be traced to 2 facts: (1) the historical precedent of placing too much reliance on an unnecessary limited set of prognostic variables (eg, lymph node status) for the purposes of stratifying patients and making therapeutic decision and (2) the impossibility of determining the actual nature of a high proportion of mesenteric deposits [4,7]. Herein we took both of these factors into account and recommend that in situations in which small mesenteric and perirectal tumor deposits might be diagnosed as lymph nodes, the total number and the size of the largest deposits should be recorded and the clinician be made aware that these lesions are likely to be associated with an adverse prognosis.

## Conclusion

These results suggest that standard pathologic examination underestimates the number of metastases in mesocolon specimens from colorectal cancer patients by failing to detect not only small involved nodes but also extramural metastases. Routine pathologic examination should focus on these foci of cancer metastasis and help to refine the staging of patients with colorectal cancer. The value of this finding for improving the TNM classification, together with the classic prognostic factors, will provide a more accurate classification, benefit adjuvant chemotherapy for patients and assess more exact prognostic information.

## Competing interests

The authors declare that they have no competing interests.

## Authors' contributions

The work presented here was carried out in collaboration between all authors. RHX and ZZW defined the research theme and research methods. HBQ and GC co-worked on associated data collection, interpretation and discussed analyses and wrote the paper, RPK revised the manuscript, HYL and MZQ analyzed the data and interpreted the results. FW evaluated the pathological data. All authors have contributed to, seen and approved the manuscript.

## Pre-publication history

The pre-publication history for this paper can be accessed here:

http://www.biomedcentral.com/1471-2407/11/414/prepub
